# Metastatic-prone telomerase reverse transcriptase (*TERT*) promoter and v-Raf murine sarcoma viral oncogene homolog B (*BRAF*) mutated tall cell variant of papillary thyroid carcinoma arising in ectopic thyroid tissue: A case report

**DOI:** 10.1097/MD.0000000000024237

**Published:** 2021-01-15

**Authors:** Adam Stenman, Anna Koman, Catharina Ihre-Lundgren, Carl Christofer Juhlin

**Affiliations:** a Department of Oncology-Pathology; b Department of Molecular Medicine and Surgery, Karolinska Institutet; c Department of Breast, Endocrine Tumors and Sarcoma; d Department of Pathology and Cytology, Karolinska University Hospital, Stockholm, Sweden.

**Keywords:** case report, ectopic thyroid, papillary thyroid carcinoma, telomerase reverse transcriptase, v-Raf murine sarcoma viral oncogene homolog B

## Abstract

**Rationale:**

Mutations of the v-Raf murine sarcoma viral oncogene homolog B *(BRAF)* oncogene and telomerase reverse transcriptase (*TERT*) promoter region are indicators of poor prognosis in papillary thyroid carcinoma (PTC) and might predict future occurrences of distant metastases. However, the clinical significance of these genetic aberrancies in PTCs arising in ectopic locations is not well established.

**Patient concerns:**

We describe a patient with a previous history of radioiodine (RAI)-treated hyperthyroidism and a surgically resected right-sided follicular thyroid adenoma. In 2013, a 6 mm follicular variant papillary thyroid carcinoma was diagnosed following a left-sided thyroid lobectomy. The central compartment displayed 9 tumor-free lymph nodes, and no adjuvant treatment was planned.

**Diagnoses:**

Three years later, a 26 mm pre-tracheal relapse was noted, however, the excised lesion was consistent with a tall cell variant of papillary thyroid carcinoma (TCV-PTC) arising in ectopic thyroid tissue. RAI treatment was commenced. Four years later, a 5 mm subcutaneous lesion in the anterior neck was surgically removed and diagnosed as metastatic TCV-PTC with a codon 600 *BRAF* mutation and a C228T *TERT* promoter mutation.

**Interventions:**

RAI treatment was re-initiated. Molecular re-examination of the primary follicular variant papillary thyroid carcinoma demonstrated a codon 600 *BRAF* mutation and a *TERT* promoter wildtype sequence, while the primary TCV-PTC was positive for mutations in both codon 600 of *BRAF* as well as the *TERT* promoter.

**Outcomes:**

The patient is alive and well without signs of relapse 7 months after the latest round of RAI.

**Lessons:**

We conclude that the occurrence of combined *BRAF* and *TERT* promoter mutations in the primary lesion from 2016 was associated to the manifestation of distant metastases 4 years later, strengthening the benefit of mutational screening of these genes in clinical routine for thyroid carcinomas arising in aberrant locations.

## 1 Introduction

Ectopic cervical thyroid tissue is an uncommon finding in the clinical setting with an estimated prevalence of 1:10,000 individuals.^[1–3]^ The development of a primary thyroid carcinoma in extrathyroidal locations is even more exceptional, and hence the scientific community is largely dependent on single case reports or small case series in order to draw conclusions regarding how to diagnose and treat these lesions.^[4]^ As a consequence, treatment options and follow-up schemes for these lesions probably vary across different institutions. The most common sites of ectopic cervical thyroid tissue are lingual, sublingual, thyroglossal, laryngo-tracheal and lateral cervical compartments, and believed to be a result of aberrant embryonic migration from the pharyngeal pouches.^[2,4]^

Although rare, the possibility of tumor development in an ectopic location is frequently discussed in the clinical setting, as the distinction between a primary thyroid carcinoma arising in ectopic thyroid tissue and a cervical lymph node metastasis is not always easily determined. This is especially true in the preoperative setting using fine-needle aspiration biopsy (FNAB) examination of lesions separated from the thyroid gland, when the thyroid itself is free of visible tumor masses from a radiological perspective. Although rare, whenever a thyroid tumor develop in ectopic thyroid tissue, findings of papillary thyroid carcinomas (PTCs) dominate, though follicular thyroid carcinomas and occasional reports of medullary thyroid carcinoma and poorly differentiated thyroid carcinoma also exist.^[4,5]^ Interestingly, different ectopic locations seem to confer a predilection for certain tumor types; for example, lingual thyroid tissue most often harbor follicular thyroid carcinomas, whereas thyroid carcinoma arising in a thyroglossal duct cyst most often are PTCs, and rarely squamous cell carcinoma or anaplastic thyroid carcinoma.^[4]^

Thyroid carcinomas arising in ectopic thyroid tissue cannot be risk stratified as conventional tumors, and predictive factors such as extrathyroidal extension and the pathological tumor node and metastasis (TNM) staging are redundant for these cases. Therefore, practicing clinicians are recommended to make assessment based on individual risk stratifications.^[4,6]^ While surgical removal seems to be the most appropriate treatment option for ectopic primary thyroid cancer of the cervical compartment, the beneficial use of postoperative radioiodine (RAI) is not established. Moreover, little is known regarding the risk of metastatic spread and what factors that predict aggressive biological features.

In this report, we describe the clinical, histological, and molecular features of a PTC arising in pre-laryngeal, ectopic thyroid tissue, and the subsequent development of subcutaneous metastatic deposits. More specifically, we highlight how v-Raf murine sarcoma viral oncogene homolog B (*BRAF*) and telomerase reverse transcriptase (*TERT*) promoter analyses of PTCs arising in ectopic thyroid tissue might be a beneficial strategy to pinpoint cases at risk for recurrences similar to the established risk stratification used for tumors arising within the thyroid gland itself.

## 2 Case report

The patient is a 69-year old female without family history indicating thyroid disorders. After being diagnosed with a follicular thyroid tumor 30 years ago, the patient underwent a right-sided hemithyroidectomy, and the ensuing histopathology report described a follicular thyroid adenoma. Five years after the initial hemithyroidectomy, she was diagnosed with thyrotoxicosis treated with low-dose RAI followed by thyroid-hormone replacement. In 2013, at the age of 61, she noticed a small swelling at the left side of the neck and was referred to our endocrine surgery department. Neck ultrasound showed a 15 mm lesion in the left thyroid lobe and a 10 mm large, right-sided cervical lymph node in level II, and she subsequently underwent FNAB of both masses. The cytology report of the left-sided thyroid lesion described papillary formations displaying enlarged nuclei with pseudo-inclusions and grooves, and immunocytochemistry demonstrated positivity for thyroid transcription factor 1 (TTF1), paired box gene 8, and thyroglobulin. The diagnosis was a PTC (Bethesda VI). FNAB of the 10 mm lymph node in level 2 could not pinpoint metastatic disease.

**Figure 1:**
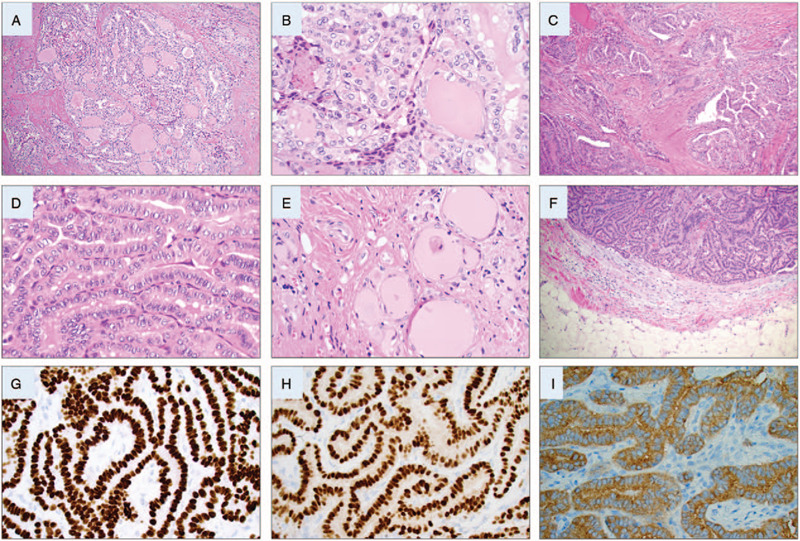
Histological and immunohistochemical attributes of the diagnosed tumors. All photomicrographs are magnified ×400 if not otherwise specified. (A) Routine hematoxylin-eosin (H&E) staining of the 6 mm follicular variant papillary thyroid carcinoma (FV-PTC) in the left thyroid lobe, demonstrating a micro-follicular growth pattern and lack of encapsulation. Magnification ×100. (B) Same tumor displaying all characteristic PTC associated nuclear features, such as enlarged tumor nuclei with irregular contours and chromatin clearing as well as occasional nuclear membrane features such as grooves and pseudo-inclusions. (C) H&E staining of the pre-laryngeal lesion magnified ×100, displaying a papillary tumor with an infiltrative growth pattern in a sclerotic stroma. The lesion was not structured as a lymph node metastasis, and no lymphoid tissue was present. (D) Same tumor demonstrating taller-than-wide cells with an abundant eosinophilic cytoplasm and PTC related nuclear features, consistent with a tall cell variant papillary thyroid carcinoma (TCV-PTC). (E) Remnants of thyrocytes without atypia and central colloid deposits in the periphery of the TCV-PTC strongly argued in favor for ectopic thyroid tissue and against a lymph node metastasis. The features of this ectopic normal thyroid tissue are further detailed in Figure 2. (F) Subcutaneous metastasis of a TCV-PTC 4 yr later, magnification ×100. (G–I) Immunohistochemistry was positive for TTF1, PAX8, and codon V600 mutation specific *BRAF* protein respectively, proving thyroid origin. *BRAF* = v-Raf murine sarcoma viral oncogene homolog B, PAX8 = paired box gene 8, TTF1 = thyroid transcription factor 1.

The patient underwent left-sided hemithyroidectomy and central neck dissection 2 weeks later. The thyroid lobe weighted 1.6 g. and measured 28 × 18 × 9 mm. By gross pathology investigation, a 6 mm large lesion was evident in the central aspects of the lobe and submitted entirely for microscopy. The histopathological investigation displayed a poorly circumscribed, follicular-patterned tumor growing infiltrative in the surrounding thyroid parenchyma. Tumor nuclei were enlarged and displayed crowding, nuclear grooves, loose chromatin, and occasional pseudo-inclusion bodies (Fig. 1A and B). The features were consistent with a follicular variant papillary thyroid carcinoma (FV-PTC). No extrathyroidal extension was observed, and surgical margins were tumor-free. Immunohistochemical analyses displayed positivity for the V600E mutation specific *BRAF*, and the Ki-67 proliferation index was 1%. For the former staining, we employed the Ventana anti-*BRAF* V600E mouse monoclonal antibody (clone VE1) that specifically targets the V600E mutation, and thus fails to recognize wildtype protein at this codon. To obtain a Ki-67 index, we count at least 2000 tumor cells in hot spot regions (defined as areas with the highest proportion of Ki-67 positive cells), dividing the number of positive nuclei with the total amount of tumor nuclei manually using an ocular with a counting grid. Nine tumor-free lymph nodes were found in the central compartment. The pTNM was pT1aN0, and the patient was discharged from further surveillance without adjuvant RAI treatment.

**Figure 2:**
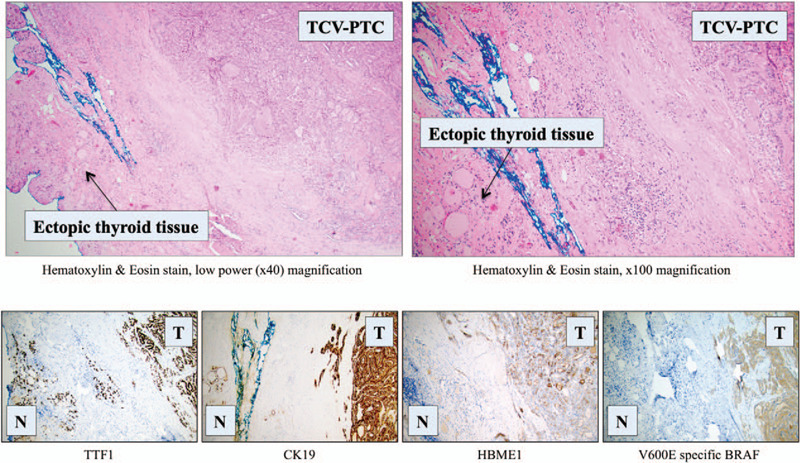
Extended histological and immunohistochemical features of the ectopic pre-laryngeal thyroid tissue. Histologically normal thyroid tissue is evident in the periphery of the specimen, bordering the blue ink of the resection margin. Note the adjacent tall cell variant of papillary thyroid carcinoma (TCV-PTC). Using immunohistochemistry, the ectopic normal thyroid tissue (N) was diffusely positive for TTF1 and focally positive for CK19, while negative for HBME1 and V600E specific *BRAF*. In contrast, note the distinct tumoral expression (T) for all markers. The normal tissue was also positive for thyroglobulin (data not shown). All immunohistochemical stains are magnified ×100. *BRAF* = v-Raf murine sarcoma viral oncogene homolog B, CK19 = cytokeratin 19, HBME1 = Hector Battifora mesothelial-1, TTF1 = thyroid transcription factor 1.

Three years later, in 2016 she was referred to endocrine surgery department again due to a swelling at the left side of the neck. Ultrasound showed a 13 mm pre-laryngeal lesion, which was also visualized using an iodine-131 scintigraphy. An ensuing FNAB was again consistent with PTC (Bethesda IV). The pre-laryngeal lesion was resected shortly thereafter. The specimen displayed a weight of 3 g, measured 26 × 16 × 13 mm, and the lesion was submitted entirely for microscopy. The ensuing investigation displayed a well-demarcated tumor with a papillary growth pattern against a background of a sclerotic stroma (Fig. 1C). The tumor cells displayed oncocytic-like cytoplasmic features and were 2 to 3 times as tall as they were wide in >30% of the tumor cell proportion (Fig. 1D). Typical nuclear features of PTC were present, and the cells were positive for thyroglobulin, TTF1, cytokeratin 19 (CK19), Hector Battifora mesothelial-1 (HBME1), and V600E mutation specific *BRAF* (Fig. 2). The Ki-67 proliferation index was 6%. In peripheral areas of the lesion, follicular-patterned tissue without nuclear features of PTC was observed, diffusely positive for thyroglobulin and TTF1, partially positive for CK19, but negative for HBME1 and V600E mutation specific *BRAF* – consistent with normal thyroid tissue (Figs. 1E and 2). The final diagnosis was a tall cell variant of papillary thyroid carcinoma (TCV-PTC) arising in pre-laryngeal, ectopic thyroid tissue. The lesion was excised with negative margins. The patient was discussed at a multidisciplinary conference, where adjuvant RAI treatment of 3,7 Giga-Becquerel was recommended and thereafter administered.

The patient was free of relapse until January 2020, when an 8 × 4 mm subcutaneous lesion was noted just ventral to the trachea via ultrasonography of the anterior neck. The lesion raised the suspicion of a metastasis and was subsequently resected. The skin-covered specimen measured 33 × 11 × 10 mm and was sectioned by the surgical pathologist and submitted in whole for histopathological investigations. By light microscopy, a subcutaneous manifestation of a tumor with predominant papillary to focally cystic growth patterns was observed. Moreover, PTC-associated nuclear changes and a tall cell phenotype were evident (Fig. 1F). Intravascular tumor deposits were noted. By immunohistochemistry, the metastasis was positive for TTF1, paired box gene 8, CK19, HBME1, and V600E mutation specific *BRAF* (Fig. 1G–I). Moreover, weak immunoreactivity for thyroglobulin was noted in approximately 25% of tumor nuclei. The Ki-67 proliferation index was 7.2%. Surgical margins were negative. Metastatic TCV-PTC was diagnosed. The patient was subsequently treated with 3,7 Giga-Becquerel RAI postoperatively, and is currently alive and well without signs of relapse. Her latest serum thyroglobulin count was <0.1 micrograms/liter (μg/L) (reference: <1.0 μg/L), with a thyroglobulin antibody count of <1.0 kilo-units (kU)/L (reference <4.0 kU/L), and a whole-body scintigraphy with radioiodine-131 showed no aberrant findings.

Given the rather unusual presentation, molecular examinations of all 3 PTCs were initiated. DNA was extracted via tissue cut from a representative formalin-fixated paraffin-embedded block using the Maxwell 16 formalin-fixated paraffin-embedded Tissue LEV DNA kit (Promega, Madison, WI). For the *BRAF* V600 mutational screening, we used the Idylla *NRAS-BRAF* Assay (Biocartis, Mechelen, Belgium). This assay screens for NRAS proto-oncogene GTPase (*NRAS)* gene mutations in exon 2, 3, and 4 as well as mutations in exon 15 of the *BRAF* gene (codon 600; V600E, V600D, V600K, and V600R) using a real-time PCR based methodology. The methodology regarding Sanger sequencing of the *TERT* promoter has been previously described in detail.^[7]^ The 6 mm FV-PTC diagnosed in the left thyroid lobe was found to display a codon 600 *BRAF* mutation but negative for *TERT* promoter mutations as well as hotspot *NRAS* mutation of exons 2, 3, and 4. Both the pre-laryngeal TCV-PTC and the ensuing subcutaneous metastasis were positive for mutations in both *BRAF* codon 600 and the C228T *TERT* promoter mutation. Moreover, both these lesions were *NRAS* wildtype (Table 1).

**Table 1:** Molecular and immunohistochemical aberrations of the resected tumor specimen.

Year of surgery	Tumor type	*BRAF* status	*NRAS* status	*TERT* promoter status	Ki-67 index
2013	FV-PTC	V600 mutation	Wildtype	Wildtype	1%
2016	TCV-PTC	V600 mutation	Wildtype	C288T mutation	6%
2020	Met TCV-PTC	V600 mutation	Wildtype	C288T mutation	7.2%

*BRAF* = v-Raf murine sarcoma viral oncogene homolog B, FV-PTC = follicular variant papillary thyroid carcinoma, Met = metastatic, TCV-PTC = tall cell variant papillary thyroid carcinoma, *TERT* = telomerase reverse transcriptase.

The study was approved by the Swedish Ethical Review Authority (approval no. 2015/959-31), and informed consent was obtained from the patient.

## 3 Discussion

In this report, we provide evidence that ectopically derived PTC could harbor established mutations in genes characteristically associated to worse clinical outcome, namely *BRAF* and the *TERT* promoter.^[7–12]^ Mutations in these genes are known to collectively highlight PTC specimen at risk of future recurrences. Indeed, in our case, the detection of synchronous manifestation of mutations in these genes was associated to the occurrence of distant metastases 4 years later.

The development of a carcinoma in ectopic thyroid tissue mandates several clinically relevant considerations, not least to pinpoint whether the tumor developed de novo or in fact constituted a metastasis from a primary tumor within the thyroid gland itself. In our case, the detection of a histologically normal thyroid remnant at the periphery of the excised lesion strongly argued in favor for the former rather than the latter assumption (Fig. 2). The question also arose whether or not the TCV-PTC described herein in fact developed from an unidentified remnant of the pyramidal lobe than in bona fide ectopic thyroid tissue. However, this was deemed unlikely given the previous radiological and operation reports that did not mention any observation of a pyramidal lobe or indicating visible remnant thyroid tissue. As of such, the tumor was assumed to arise in ectopic laryngotracheal thyroid tissue. Indeed, this is one of the most frequent sites of ectopic thyroid tissue when consulting previous literature.^[4]^ Moreover, the possibility that the tumor developed within a thyroglossal duct cyst was excluded, as the lesion was void of cystic features and unassociated to the hyoid bone.

The patient was diagnosed with a follicular thyroid adenoma 30 years ago, first by cytology using a fine-needle aspiration biopsy and subsequently also by histopathology following a hemithyroidectomy. Unfortunately, as this surgery was performed in another hospital, we were unable to retrieve these slides for review purposes. As of this, the chance of this tumor actually being a misdiagnosed FV-PTC was considered. Even so, a rendered FV-PTC diagnosis should have little impact upon the conclusions of this study - as the metastatic-prone PTC in our case was a TCV-PTC without the morphological characteristics of an FV-PTC. Moreover, we do not believe that the occurrence of an FV-PTC in the thyroid gland and a separate TCV-PTC in ectopic thyroid tissue would indicate a genetic association, but rather a coincidence given the high incidence of PTC in the general population. However, we cannot fully exclude environmental factors as contributors of the disease presentation. In general, FV-PTCs are clinically indolent *RAS*-driven tumors, while TCV-PTCs are *BRAF*-driven cancers with poor prognosis, and therefore no apparent genetic association exist – even though the FV-PTC in our case was *BRAF* mutated, which is unusual but not entirely uncommon.^[13]^

Previous literature suggest that thyroid carcinomas occurring in ectopic cervical locations not seldom give rise to lymph node metastases, thereby potentially arguing that the malignant potential of these lesions demand careful risk assessments.^[14,15]^ As the practicing clinicians cannot rely on standard prognostic parameters such as tumor size and the TNM staging when assessing thyroid carcinomas arising in an aberrant location, the detection of genetic aberrancies with a known coupling to worse outcome might be useful in determining the future surveillance and/or adjuvant treatment options for these rare patients. In fact, genomic testing as a diagnostic and predictive marker in advanced well-differentiated thyroid carcinomas is nowadays recommended for subsets of cases according to The National Comprehensive Cancer Network guidelines.^[16]^ The effect of *BRAF* and *TERT* promoter mutations in PTC is well established, with a strong coupling to high-risk clinico-pathological variables such as extrathyroidal extension, TNM stage, lymph node, and distant metastases in addition to worse overall survival in mutated cases.^[10,11]^ While the V600E *BRAF* mutation stimulates the mitogen-activated protein kinase signaling cascade and results in increased tumor growth and oncogenic transformation, the C228T and C250T mutations of the *TERT* promoter stimulates *TERT* gene expression, thereby presumably immortalizing the cells through the telomerase driven effects on telomere length.^[17,18]^ Of particular interest, the coexistent occurrence of *BRAF* and *TERT* promoter mutations in PTCs seem to exhibit synergistic effects on clinical outcomes.^[10,11]^ Interestingly, both V600E *BRAF* mutations as well as *TERT* promoter mutations are overrepresented in TCV-PTCs compared to many other variants of PTCs, including classical variant PTCs, possibly providing a molecular explanation for the overall poorer prognosis in TCV-PTCs.^[12,19]^ As both mutations are tightly coupled to patient outcome, sequencing of these variants are often used in single-gene, clinical routine screening programs as well as in comprehensive gene panels for both clinical and research purposes.^[7,20–22]^ The single-gene analyses are cheap, reproducible, and easily interpretable, providing a possibility to screen thyroid tumors even in laboratories lacking state-of-the-art next generation sequencing equipment. The finding of both a codon 600 *BRAF* and a C228T *TERT* promoter mutation in the TCV-PTC arising ectopically in our case mirrors previous observations in high-risk cases of thyroid tumors arising within the thyroid gland, as our patient developed distant metastases 4 years after surgical removal. Therefore, if these genetic events would have been known to the clinician at the time of the surgical excision, adjustments to the administered postoperative RAI dose and/or follow-up scheme would possibly have been adequate – even though our patient exhibited a risk profile irrespectively of molecular data given her age (>55 years at the time of diagnosis) and the occurrence of a histological PTC subtype frequently associated to worse clinical outcome.

A limitation to the current study is the fact that the Idylla *NRAS-BRAF* Assay, although highly sensitive and specific, do not discriminate between *BRAF* V600E and V600D mutations. Thus we cannot with certainty state that the *BRAF* mutations found herein were identical. However, in PTCs, the V600E mutation is by far the most common mutation type – and as the *BRAF* V600E mutation specific immunohistochemistry was also clearly positive for all described tumors, the chance of these *BRAF* codon V600 mutations being disparate is minimal. Another drawback to our study design is the lack of *TERT* gene expressional data in the *TERT* promoter mutated tumors, which was due to the lack of fresh frozen material for mRNA analyses. Although antibodies are available for the immunohistochemical detection of the TERT protein, our previous experiences suggest that the latter method is not yet a reliable tool, at least not for follicular thyroid tumors.^[23]^

We conclude that this TCV-PTC arising in ectopic laryngotracheal thyroid tissue did exhibit similar high-risk molecular features as counterpart tumors primary within the gland itself. As the combination of the codon V600 *BRAF* mutation and *TERT* promoter mutations has been found to predict worse outcome in PTCs in general, these molecular aberrancies could probably be of use to predict the risk of distant metastases also in PTCs arising in aberrant locations. Although these initial observations probably need to be reproduced in larger case series, the inclusion of *BRAF* and *TERT* promoter sequencing when assessing thyroid cancer from ectopic thyroid tissue might be a beneficial strategy – not least as the current recommendations are based on individualized risk assessments.

## Acknowledgments

The authors are indebted to the professional technical service of the clincial pathology team at our department, especially Dr. Kenbugul Jatta, MSc, PhD and Mr. Robin Brolin, MSc, both who have agreed upon being named in this section of the manuscript.

## Author contributions

**Conceptualization:** Catharina Ihre-Lundgren, Carl Christofer Juhlin.

**Data curation:** Adam Stenman, Anna Koman, Carl Christofer Juhlin.

**Funding acquisition:** Carl Christofer Juhlin.

**Investigation:** Adam Stenman, Anna Koman, Catharina Ihre-Lundgren, Carl Christofer Juhlin.

**Writing – original draft:** Carl Christofer Juhlin.

**Writing – review & editing:** Adam Stenman, Anna Koman, Catharina Ihre-Lundgren, Carl Christofer Juhlin.

## Abbreviations

Abbreviations: μg = microgram, CK19 = cytokeratin 19, FNAB = fine-needle aspiration biopsy, FV-PTC = follicular variant papillary thyroid carcinoma, HBME1 = hector battifora mesothelial-1, kU = kilo-units, L = liter, NRAS = NRAS proto-oncogene GTPase, PTC = papillary thyroid carcinoma, RAI = radioiodine, TCV-PTC = tall cell variant of papillary thyroid carcinoma, TTF1 = thyroid transcription factor 1.

## References

[1] Noyek AM Friedberg J. Thyroglossal duct and ectopic thyroid disorders. Otolaryngol Clin North Am 1981;14:187–201. 7254840

[2] Santangelo G Pellino G De Falco N. Prevalence, diagnosis and management of ectopic thyroid glands. Int J Surg 2016;28: (Suppl 1): S1–6. 26708843 10.1016/j.ijsu.2015.12.043

[3] Batsakis JG El-Naggar AK Luna MA. Thyroid gland ectopias. Ann Otol Rhinol Laryngol 1996;105:996–1000. 8973289 10.1177/000348949610501212

[4] Klubo-Gwiezdzinska J Manes RP Chia SH. Clinical review: ectopic cervical thyroid carcinoma--review of the literature with illustrative case series. J Clin Endocrinol Metab 2011;96:2684–91. 21752893 10.1210/jc.2011-0611

[5] Seoane JM Cameselle-Teijeiro J Romero MA. Poorly differentiated oxyphilic (Hürthle cell) carcinoma arising in lingual thyroid: a case report and review of the literature. Endocr Pathol 2002;13:353–60. 12665653 10.1385/ep:13:4:353

[6] Haugen BR Alexander EK Bible KC. 2015 American Thyroid Association Management Guidelines for adult patients with thyroid nodules and differentiated thyroid cancer: the American Thyroid Association guidelines task force on thyroid nodules and differentiated thyroid cancer. Thyroid 2016;26:1–33. 26462967 10.1089/thy.2015.0020 PMC4739132

[7] Hysek M Paulsson JO Jatta K. Clinical routine TERT promoter mutational screening of follicular thyroid tumors of uncertain malignant potential (FT-UMPs): a useful predictor of metastatic disease. Cancers (Basel) 2019;11:1443. 31561592 10.3390/cancers11101443 PMC6826397

[8] Bournaud C Descotes F Decaussin-Petrucci M. TERT promoter mutations identify a high-risk group in metastasis-free advanced thyroid carcinoma. Eur J Cancer 2019;108:41–9. 30648628 10.1016/j.ejca.2018.12.003

[9] Landa I Ganly I Chan TA. Frequent somatic TERT promoter mutations in thyroid cancer: higher prevalence in advanced forms of the disease. J Clin Endocrinol Metab 2013;98:E1562–6. 23833040 10.1210/jc.2013-2383 PMC3763971

[10] Xing M Liu R Liu X. BRAF V600E and TERT promoter mutations cooperatively identify the most aggressive papillary thyroid cancer with highest recurrence. J Clin Oncol 2014;32:2718–26. 25024077 10.1200/JCO.2014.55.5094 PMC4145183

[11] Shi X Liu R Qu S. Association of TERT promoter mutation 1,295,228 C>T with BRAF V600E mutation, older patient age, and distant metastasis in anaplastic thyroid cancer. J Clin Endocrinol Metab 2015;100:E632–7. 25584719 10.1210/jc.2014-3606 PMC4399285

[12] Liu X Bishop J Shan Y. Highly prevalent TERT promoter mutations in aggressive thyroid cancers. Endocr Relat Cancer 2013;20:603–10. 23766237 10.1530/ERC-13-0210 PMC3782569

[13] Cancer Genome Atlas Research Network. Integrated genomic characterization of papillary thyroid carcinoma. Cell 2014;159:676–90. 25417114 10.1016/j.cell.2014.09.050 PMC4243044

[14] Kennedy TL Riefkohl WL. Lingual thyroid carcinoma with nodal metastasis. Laryngoscope 2007;117:1969–73. 17828053 10.1097/MLG.0b013e31812e0160

[15] Massine RE Durning SJ Koroscil TM. Lingual thyroid carcinoma: a case report and review of the literature. Thyroid 2001;11:1191–6. 12186508 10.1089/10507250152741055

[16] Haddad RI Nasr C Bischoff L. NCCN guidelines insights: thyroid carcinoma, version 2.2018. J Natl Compr Canc Netw 2018;16:1429–40. 30545990 10.6004/jnccn.2018.0089

[17] Caronia LM Phay JE Shah MH. Role of BRAF in thyroid oncogenesis. Clin Cancer Res 2011;17:7511–7. 21900390 10.1158/1078-0432.CCR-11-1155

[18] Colebatch AJ Dobrovic A Cooper WA. TERT gene: its function and dysregulation in cancer. J Clin Pathol 2019;72:281–4. 30696697 10.1136/jclinpath-2018-205653

[19] Dettmer MS Schmitt A Steinert H. Tall cell papillary thyroid carcinoma: new diagnostic criteria and mutations in BRAF and TERT. Endocr Relat Cancer 2015;22:419–29. 25870252 10.1530/ERC-15-0057

[20] Decaussin-Petrucci M Descotes F Depaepe L. Molecular testing of BRAF, RAS and TERT on thyroid FNAs with indeterminate cytology improves diagnostic accuracy. Cytopathology 2017;28:482–7. 29094776 10.1111/cyt.12493

[21] Endo M Nabhan F Porter K. Afirma gene sequencing classifier compared with gene expression classifier in indeterminate thyroid nodules. Thyroid 2019;29:1115–24. 31154940 10.1089/thy.2018.0733 PMC7141558

[22] Nikiforov YE Baloch ZW. Clinical validation of the ThyroSeq v3 genomic classifier in thyroid nodules with indeterminate FNA cytology. Cancer Cytopathol 2019;127:225–30. 30811896 10.1002/cncy.22112 PMC6519348

[23] Paulsson JO Olander A Haglund F. TERT immunohistochemistry is a poor predictor of TERT promoter mutations and gene expression in follicular thyroid carcinoma. Endocr Pathol 2018;29:380–3. 30306386 10.1007/s12022-018-9551-6 PMC6223712

